# Nutritional Status Assessment of Minodar Residence in Qazvin City, Iran: Vitamin D Deficiency in Sunshine Country, a Public Health Issue

**DOI:** 10.5539/gjhs.v5n1p174

**Published:** 2012-11-21

**Authors:** Amir Ziaee, Amir Javadi, Maryam Javadi, Mohammadali Zohal, Ahmad Afaghi

**Affiliations:** 1 Qazvin Metabolic Diseases Research Center, Qazvin University of Medical Sciences, Qazvin, Iran; 2 Community Medicine, School of Medicine, Qazvin University of Medical Sciences, Qazvin, Iran; 3 Department of Nutrition, School of Health, Qazvin University of Medical Sciences, Qazvin, Iran; 4 Department of Internal Medicine, School of Medicine, Qazvin University of Medical Sciences, Qazvin, Iran; 5 Qazvin Research Center for Social Determinants of Health Science (QRC SDH), Qazvin University of Medical Sciences, Qazvin, Iran

**Keywords:** nutrition assessment, energy, mineral, vitamin D

## Abstract

**Introduction:**

Nutrition has main effect on health or disease and results of nutrition assessment can be used in health planning of communities. Therefore we aimed to conduct the nutrition assessment especially vitamin D statue of an urban region in Qazvin city.

**Methods:**

In a cross sectional study in year 2011, subjects who were randomly selected from residents of aged ≥ 20 years old in Minodar, an urban region of Qazvin city participated in this study. A 3-days food diary questionnaire was used to collect food consumption data and weight and height of subjects were measured. The food intake was analysis using “Nutrition 4, Diet analysis, Module version 3.5.2”.

**Results:**

The participants included 930 subjects (434 M and 496 F) having Mean BMI = 26 ± 4.4 kg/m^2^ and 60% of subjects were either overweight or obese. Daily energy intake of 50% of subjects was more than 2500 (13.6% from proteins, 55.6% from carbohydrate and 30.5% from fat sources). The daily cholesterol intake among 50-75% of population was more than daily recommended of 300 mg. The mean iron and zinc intakes were 17±5.6 and 12±4.5 mg/day and the intakes were highest in aged group of 20-29, while reduced in older groups. Vitamin A deficiency was observed in studied population and 75% of them were receiving less than daily recommended allowance of 800 μg/day, 25% of total studied population was receiving less than 400 μg/day. Vitamin D ingestion among 90-95% of participants was less than minimum daily recommended amount of 10 μg/day and calcium intake in 50-75% of studied population was less than recommended daily allowance of 800 mlg/day. High amount of florid and caffeine ingestion from black tea was observed among population and 75% of population had florid intake of 10000 mμ/day.

**Conclusion:**

In general, majority of the population of region had higher amount intake of fat, cholesterol, low intake of calcium and vitamin D

## 1. Introduction

Nutrition has main effect on health or disease and results of nutrition assessment can be used in health planning of communities. Micronutrients deficiencies such as vitamin A, D, Ca^2+^ and Iron have been demonstrated in different countries including Iran. In previous study (ERC Accessed Sep.2012) in Tehran population it was demonstrated that 30% of energy intake was from fat sources and cholesterol intake was higher than RDA recommendation of 300 mg. The Ca^2+^ and Iron intake were lower than RDA recommendation (Mahan and Escott-Stump, 2012).

Among micronutrient the vitamin D deficiency is now recognized as a pandemic. The major cause of vitamin D deficiency is the lack of moderate sunlight exposure. Limited animal sources of foods are rich in vitamin D, and foods that are fortified with vitamin D are often are not enough to provide population needs. Vitamin D deficiency causes rickets in children and leads to osteopenia, osteoporosis, and consequently bone fractures in adults. Vitamin D deficiency has been associated with increased risk of common cancers, autoimmune diseases, hypertension, and infectious diseases (Holick and Chen 2008). The relation between vitamin D deficiency and heart disease has been demonstrated in different studies (Sun et al., 2011)

In China, the prevalence of vitamin D deficiency investigated using serum 25(OH)D among 6008 children of 1 month to 16 years old in Hangzhou (latitude: 30°N). In winter and spring, more than 50% of school age children and adolescents had a 25(OH)D level at < 50 nmol/L. If the threshold is changed to < 75 nmol/L, all of the adolescents (100%) and 93.7% of school age children had low 25(OH)D levels in winter (Zhu et al., 2012). In randomly assigned 540 urban/rural centers in Turkey Approximately 60% of the population had severe/moderate Vitamin-D deficiency (Satman et al., 2012). According to different studies that have been conducted across India, there are widespread prevalence of varying degrees (50- 90%) of Vitamin D deficiency with low dietary calcium intake in Indian population (Londhey, 2011) and in Iran the moderate to sever vitamin D deficiency was reported between 37.5-54.5% among adults in multi-center studies (Heshmat et al., 2008). Also, the prevalence of moderate to mild vitamin D deficiency in a cross sectional study in year 2010 among 420 (220 girls, 200 boys) secondary school students in Arak city (Iran) was 22.4 and 60.7% respectively (Talaei et al., 2012).

In study conducted by Moradzadeh et al. (2008), the prevalence of vitamin D deficiency among adults of 20-29 years old in 5 regions of Iran with different climate has been reported as 75% and 74% in women and men respectively. In another study carried out by Heshmat et al. (2008) in a random cluster sample of 5232 men and women in 5 major cities of Iran in year 2001 (Tehran, Tabriz, Mshhad, shiraz, Boshehr), the prevalence of vitamin D deficiency in men and women were 44.2-47.2% and 37.5%-54.2% respectively. The highest prevalence was observed in Tehran while the lowest was observed in south of Iran, Bosher which was assumed to be due to heterogeneity in Bosher region. Also Hashemipour et al. (2004) reported that, the sever, moderate and mild vitamin D deficiency in Tehranian population was 9.5%, 57.6% and 14.2% respectively. Different studies in Iran (Hashemipour et al., 2004; Rahnavard et al., 2010) did not observe any relation between blood serum vitamin D level and time exposure to sunlight, however they found the geographic area as independent factor for blood serum vitamin D level (Rahnavard et al., 2010).

Anecdotal studies demonstrated that there are wide spread blood serum vitamin D concentration deficiency in Qazvin region. Therefore we aimed to conduct the nutrition assessment of an urban region in Qazvin city to find out the macro and micro nutrient intake specifically Vitamin D ingestion.

## 2. Methods

In a cross sectional study, randomly selected subjects from residents of aged ≥ 20 years old in Minodar, an urban region of Qazvin city (Iran) participated in this study. A 3-days Food diary questionnaire was used to collect food consumption data and weight and height of subjects were measured. Subjects who were consuming supplement excluded from the study. The food intake were analyzed using “Nutrition 4, version 3.5.2” software (Nutrition 4, 2011). The research proposal was approved by Ethical Committee of Qazvin University of Medical Sciences.

## 3. Results

The participants included 930 subjects (434 M and 496 F) with their age groups in Table 1. The study population had mean BMI = 26 ± 4.4 kg/m^2^ and 60% of subjects were either overweight or obese. Daily energy intake of 50% of subjects was more than 2500 kcal (13.6% proteins, 55.6% carbohydrate and 30.5% from fat sources) having 20 gr. daily fiber (Table 2). The daily cholesterol intake among 50-75% of population was more than daily recommended of 300 mg. (Table 2). The mean iron and zinc intakes were 17±5.6 and 12±4.5 mg/day respectively and the intakes were highest in aged group of 20-29, while reduced in older groups (Table 3). Vitamin A intake deficiency was observed in studied population and 75% of them were receiving less than daily recommended allowance of 800 μg/day, and 25% of total studied population was receiving less than 400 μg/day vitamin A (Table 4). Vitamin D ingestion among 90-95% of participants was less than the minimum daily recommended amount of 10 μg/day (Table 4) and calcium intake in 50-75% of studied population was less than recommended daily allowance of 800 mg/day (Table 3). High amount of florid and caffeine ingestion from black tea was observed among population and 75% of population had florid intake of more than 10000 μg/day.

**Table 1:** Age group of studied population

Age group	No	Percent
20-29	164	17.6
30-39	225	24.2
40-49	399	42.9
50-59	104	11.2
60-69	27	2.9
>=70	11	1.2
Total	930	100

**Table 2:** Energy, macronutrient and fiber intake in studied population

		Energy Kcal	CHO gr	Cholesterol mg	Protein gr	Fat gr	Fiber gr	Protein%	CHO%	Fat%	BMI Kg/m^2^
	No	929	930	930	930	930	930	930	930	926	930
Mean		2591	364	306	89	89	20	13.6	55.6	30.5	26
SD		703	113	172	28	32	7	2.2	7.1	7	4.4
Minimum		1046	109	43	27	16	5	2	17	10	14.8
Maximum		5591	957	1299	338	359	87	30	76	58	46
	5	4416	215	109	55	45	10	44	19	18.7	11.3
	10	1797	238	132	60	53	11	47	21	20.3	12.8
	25	2098	286	183.6	70	67.3	12	51	26	23	15.5
percentile	50	2505	347	270.9	85	85	13	56	31	25.8	19.3
	75	2951	422	390	101	105.8	15	60	35	28.7	24.2
	90	3480	503	506.9	121	129	16	64.9	39	31.5	29.9
	95	3861	577	621.4	139	149.8	18	67	42	33.7	32.6

**Table 3:** Caffeine and mineral intake in studied population

			Ca mg	Zn mg	P mg	Cu mg	Mn mg	Florid μg	Caffeine mg	Fe mg	Mg mg
	No		930	930	930	930	930	930	929	930	930
Mean			733	12.5	12.5	1.7	6.6	18725.6	124.9	17	378
SD			272	4.5	4.5	----	2.2	12193.3	88.9	5.6	138.8
Minimum			133	3.3	3.3	34	1.8	2	0	5.6	120.5
Maximum			2114	7.5	70.5	10	16.5	72257	876.6	63.5	1316
percentile		5	378.5	7.5	88	7.5	3.6	3280	25	10.4	210.9
		10	433.5	8.6	1	8.6	4.1	5457	40	11.3	238.5
		25	545.9	9.9	1.2	9.9	5.1	9731	6.6	13	284.9
		50	690.8	11.7	1.5	11.7	6.2	16121	106.6	16	350.3
		75	876.6	14	1.96	14	7.8	25330	166.6	19.5	440.3
		90	1089.9	17	2.6	17	9.5	34829	225	23.9	558.2
		95	1252	19	3.1	19.6	10.6	42100	266.6	27.7	651

**Table 4:** Vitamins intake in studied population

		B6 μg	B12 μg	Vitamin D μg	Vitamin A μg	Beta carotene μg	Folic acid μg	Vitamin C mg	Vitamin E mg
No		930	930	924	914	930	930	928	930
Mean		2	3.9	4.2	858.6	1591.1	514.9	146.9	24.1
SD		------	5.8	11.6	796.4	1430.8	213.9	98.9	14.9
Minimum		62	43	3	102.6	0	35	1.6	31
Maximum		7.3	78.9	72.4	4997.3	10501	2283	570.3	112.2
percentile	5	1.1	1.6	-------	223.2	15.9	257.6	24.7	7.5
	10	1.3	1.9	-------	291.1	45.3	292.9	39.3	9.9
	25	1.6	2.3	-------	401.2	535.2	372.5	71.6	14.6
	50	2	3	--------	638.6	1231.9	483.2	126.8	20.3
	75	2.4	3.8	1.4	958	2323.8	611.6	200.1	29.7
	90	2.1	4.9	4.3	1607.3	3527.9	753.1	284.8	42
	95	3.5	6.6	31.3	2622.8	4377.1	900.7	345.1	55.9

## 4. Discussion

This study demonstrated that, daily energy intake of 50% of the studied population was more than 2500 kcal having 30.5% of energy from fat sources (Table 2). The daily cholesterol intake among 50-75% of population was more than daily recommended of 300 mg (Table 2). The mean iron and zinc intakes were 17±5.6 and 12±4.5 mg/day and the intakes were highest in aged group of 20-29, while reduced in older groups (Table 3). Vitamin A intake deficiency was observed in studied population and 75% of them were receiving less than daily recommended allowance of 800 μg/day (Table 4). The Ca^2+^ intake in 50-75% of studied population was less than recommended daily allowance of 800 mlg/day and vitamin D ingestion among 90-95% of participants was less than minimum daily recommended allowance of 10 μg/day (Table 4).

Although there are not sufficient micronutrients intakes among studied population, but vitamin D deficiency and its intakes is noticeable point. Vitamin D is an essential factor for normal metabolism of bone, bone minerals and non-bone related metabolic process. Serum vitamin D deficiency in Iran in 5 cities with different climates has been reported in 75% of women and 72.1% of men which was similar to the results of other studies in Middle East (Moradzadeh et al., 2008). In a study carried out by Hashemipour et al. (2004) in Tehran (capital of Iran), the vitamin D serum level did not have significant relation with the duration of exposure of face and hands to sunlight, kind of clothing and BMI. Vitamin D deficiency was more evidence in Tehran (Moradzadeh et al., 2008), having highest prevalence of deficiency in Tehranian men, while the lowest prevalence was reported in Mashhad (north east of Iran) and Boosher (south of Iran) among men and women. Similarly Rahnavard et al., (2010) in a study in 5 major cities reported that the Geographical zone independently predicts vitamin D status that Tehranian had highest prevalence and there was not significant association between age, physical activity and duration of exposure of face and hands to sunlight with circulating blood vitamin D levels. However those who exposed most parts of their body to the sunlight had significantly higher vitamin D level and participants who did not exposed their skin to the sunlight had significantly lower vitamin D status (Rahnavard et al., 2010). Among Iranian mothers the cord serum 25-OH D concentration was very low (4.94±9.4 nmol/l) and infants’ hypovitaminosis D were undetectable (Basir et al., 2001).

Although vitamin D intake from animal sources in our studied population was very low and 90-95% of population did intake less than RDA of 10 μg/day, but the energy intake in the region was sufficient to provide vitamin D during exposing body skin to sunlight. However, our initial biochemical and clinical study in the studied population and also biochemical analysis across country showed that the vitamin D deficiency is wide spread and even in some regions its prevalence is up to 75%. Comparison of prevalence of vitamin D deficiency in different region reveals that there may be several factors affecting on vitamin D deficiency. These factors are such as (1) air pollutions which is very high in Tehran, where the vitamin D deficiency prevalence is high. (2) Sea food consumption which is rich in vitamin D and is consumed in coastal cities such as Bosher the low prevalence vitamin D deficiency region in Iran (Rahnavard et al., 2010). (3) Clothing habits especially among women which is believed to be an important factor in vitamin D deficiency and sun barrier in some countries such as Saudi Arabia (Lips, 2007) is not the case in Iran. Similar rate of vitamin D deficiency was observed among women and men in Iran. The vitamin D deficiency among women in Bosher (south of Iran) who have high covering rate, is the least among other studied cities in Iran (Heshmat et al., 2008). The noticeable point is that the vitamin D deficiency among Iranian populations residing in other countries is prevalent (Holvik et al., 2005).

In our current study the mean fiber consumption was 20 gr a day which is indication of diet rich in vegetable sources and contains higher phytates and lower animal proteins. High phytates and low animal proteins in diets are known as a factor for vitamin deficiency (Reinhold, 1976; Smith, 1990; Finch et al., 1992). Due to drinking black tea in Iranian society, higher amount of florid intake was observed in our studied subjects. Florid may interfere with Ca^2+^ and vitamin D absorption (Goel et al., 1976).

There were limitations in our study. We did not measure blood vitamin D to compare relation between vitamin D intake and circulating blood vitamin D. We also did not measure the sunlight exposure duration of subjects to estimate the vitamin D deficiency severity among expose and non-expose subjects to sunlight.

In summary different factors affect on vitamin D deficiency including primary low intake of vitamin D, lack of exposure to sunlight, skin pigmentation, altitude of region and genetics characteristics.

Due to life style of Iranian and also Asian countries populations which results in lack of moderate exposure to sunlight, we recommend that, the regular vitamin D fortification program in national level in order to combat vitamin D deficiency as a public health issue is needed.
